# Study on Direct Synthesis of Energy Efficient Multifunctional Polyaniline–Graphene Oxide Nanocomposite and Its Application in Aqueous Symmetric Supercapacitor Devices

**DOI:** 10.3390/nano10010118

**Published:** 2020-01-08

**Authors:** Hajera Gul, Anwar-ul-Haq Ali Shah, Ulrike Krewer, Salma Bilal

**Affiliations:** 1 National Centre of Excellence in Physical Chemistry, University of Peshawar, Peshawar 25120, Pakistan; hajeragul11@yahoo.com; 2 Institute of Chemical Sciences, University of Peshawar, Peshawar 25120, Pakistan; anwarulhaqalishah@uop.edu.pk; 3 TU Braunschweig Institute of Energy and Process System Engineering, 38106 Braunschweig, Germany; u.krewer@tu-braunschweig.de

**Keywords:** PANI–GO nanocomposite, aqueous symmetric supercapacitor devices, specific capacitance

## Abstract

The synthesis of promising nanocomposite materials can always be tricky and depends a lot on the method of synthesis itself. Developing such synthesis routes, which are not only simple but also can effectively catch up the synergy of the compositing material, is definitely a worthy contribution towards nanomaterial science. Carbon-based materials, such as graphene oxide, and conjugative polymers, such as conductive polyaniline, are considered materials of the 21st century. This study involves a simple one pot synthesis route for obtaining a nanocomposite of polyaniline and graphene oxide with synergistic effects. The study was carried out in a systematic way by gradually changing the composition of the ingredients in the reaction bath until the formation of nanocomposite took place at some particular reaction parameters. These nanocomposites were then utilized for the fabrication of electrodes for aqueous symmetric supercapacitor devices utilizing gold or copper as current collectors. The device manifested a good capacitance value of 264 F/g at 1 A/g, magnificent rate performance, and capacitance retention of 84.09% at a high current density (10 A/g) when gold sheet electrodes were used as the current collectors. It also showed a capacitance retention of 79.83% and columbic efficiency of 99.83% after 2000 cycles.

## 1. Introduction

Generally, energy storage materials are classified into electrical double layer (EDL) and pseudocapacitive materials [1]. This classification is based on the charge storage mechanism. The former stores charge in the double layer at the electrolyte/electrode interface [2] while the later do this through fast faradic reaction [3]. Carbon-based materials, such as graphite, graphene, graphene oxide, and carbon nanotubes, etc., exhibit EDL capacitance while transition metal oxide or intrinsically conductive polymer (ICP) possess the pseudocapacitive behavior due to Faradic charge storage resulting from fast electron transfer reaction in the bulk of the material [4].

Among the carbon-based materials, graphene has gained remarkable attention owing to its meritorious optical and mechanical properties, excellent controllability, and manipulability [5,6]. However, most of the foreseen vital applications of graphene in aqueous environments have been restrained due to the presence of long aromatic conjugated backbones made up of an sp^2^-hybridized single layer of carbon atoms. This problem has been solved through oxidation of graphite to graphene oxide (GO), which is composed of graphene sheets with large quantities of oxygen containing functional groups, and is synthesized through the oxidation of graphite [7]. These functional groups include sp^2^-hybridized carbonyl and carboxyl groups predominantly at the edges of sheets and sp^3^-hybridized epoxide and hydroxyl groups at the at top and bottom surfaces. Van der Waals interactions among layers of GO are varied due to these functional groups, which makes them excessively hydrophilic. Due to this hydrophilic nature, its exfoliation and dispersion in aqueous media is easy. Graphene oxide or its composites have been effectively applied in energy storage devices [8,9,10]. It has the superiority of high power density, long cycling stability, and quick charge/discharge process [11]. However, due to electrical double layer mechanism, charge storage sites are only constrained to the surface and its bulk cannot be utilized, which results in low active material utilization, low conductivity, and is hampered by its energy density and specific capacitance.

Polyaniline (PANI), the most versatile member of the class of ICPs, has been applied in various energy storage devices and studied in detail [12]. The admirable potential of polyaniline as a pseudocapacitive material for energy storage lies in its easy synthesis [13], cost effectiveness, and high electric conductivity [14]. The electron transfer reaction take place in the bulk of the material, so its specific capacitance is higher than that of carbon-based materials that store charge based on the double layer mechanism. However, it has the major drawback of fading capacitance with increasing current density and cycling. This might be due to the kinetics of faradic redox reactions, which is unfavorable at higher rates. Also, during process of charge/discharge, there is a large change in the volume. PANI is synthesized through various chemical approaches to obtain various morphologies for improving functions and to enhance the performances of the assembled devices [15].

The preparation of composites through a combination of two different materials is an easy way to improve the desirable properties of the materials of interest. By proper optimization, one can achieve the goal of obtaining a composite with the best properties of the individual materials. Composite made up of carbon-based materials and PANI has been used for boosting the capacitance of supercapacitor devices. The combination of these two very important contestants for energy storage devices leads to the formation of materials that can simultaneously hold EDL and Faradic charge storage properties. Significant contemplation has been gained by composites of PANI with graphene oxide (GO), reduced graphene oxide (rGO), and graphene [16,17,18,19,20,21].

Nanostructured materials often possess unique physicochemical and electrochemical properties and find a wide range of applications in energy storage [22]. An important role in determining the properties of the synthesized materials is played by the synthesis pathway employed. Nanostructured materials with delicate and tailored properties can be obtained by designing advanced and novel routes, including, but not limited to, in situ chemical or electrochemical polymerization, solution mixing, interfacial polymerization, Pickering emulsion polymerization, and the self-assembly approach [15,23]. When the in situ polymerization method is applied, uniform dispersion of sheet materials into the polymer matrix takes place [24]. This uniform distribution is important for achieving a superior specific capacitance and excellent cycling stability during the process of charge–discharge.

In the present study, we synthesized naocomposites of PANI with GO through a simple in situ chemical oxidative pathway by adding all the reactants in a single reactor. In this study, we utilized dodecylbenzene sulfonic acid (DBSA) as a surfactant and dopant in an acidic environment of aqueous sulfuric acid to obtain PANI–GO nanocomposite. DBSA is a bulky molecule and its incorporation minimizes the rigidity of the PANI backbone and makes the PANI more granular, dense, and porous as compared to PANI without DBSA [25]. The presence of the dodecyl (C_12_H_25_) group offers non-polar interactions with the surrounding aqueous medium and it resists the composite material to phase out. The presence of sulfuric acid not only provides a proper acidic environment for the formation of radical cations but it also acts as a dopant. Controlled synthesis is very sensitive to the reaction parameters. Zhang et al. [26] found that composites of GO with PANI show higher conductivity when a small amount of GO was used. Therefore, we varied the percentage of GO during synthesis to achieve the best optimization. The optimized sample was then utilized as the electrode material for aqueous symmetric supercapacitor device fabrication, with the motivation of establishing an electrochemical energy storage device that has enhanced energy density, stability during charge discharge cycles, and higher rate capabilities. To the best of the author’s knowledge, there is no report available on direct one pot synthesis of PANI–GO nanocomposite, simultaneously doped with DBSA and sulfuric acid, and its utilization as an electrode material for symmetric ultracapacitor assembly.

## 2. Material and Methods

### 2.1. Materials

Chloroform (CHCl_3_), dodecylbenzenesulfonic acid (DBSA) C_12_H_25_C_6_H_4_SO_3_H, aniline (C_6_H_5_NH_2_), sulfuric acid (H_2_SO_4_), ammonium persulfate ((NH_4_)_2_S_2_O_8_), graphite powder, potassium permanganate (KMnO_4_), hydrogen peroxide (H_2_O_2_), sodium nitrate (NaNO_3_), hydrochloric acid (HCl), N,N-dimethylformamide (DMF), polytetrafluoroethylene (PTFE), and acetone were purchased from Sigma-Aldrich (Hamburg, Germany) and used as received.

### 2.2. Synthesis of Graphene Oxide Sheets

The graphene oxide (GO) was synthesized by the modified Hummer’s method [27]. Accordingly, 46 mL of H_2_SO_4_ and 2.0 g of graphite powder were mixed and stirred for 2 h at 20 °C. After this, 6 g of KMnO_4_ and 1 g of NaNO_3_ were added to this stirring mixture. The temperature was then increased to 35 °C for 30 min. To this suspension, 92 mL of deionized water were slowly added, which generated violent effervescence. The temperature was controlled at 98 °C for 15 min. The mixture was diluted with 280 mL of deionized water followed by the addition of 30% H_2_O_2_ (10 mL) in order to reduce the unreacted permanganate. Five percent HCl aqueous solution was used for washing the suspension while the residual salts were removed with plenty of deionized water.

### 2.3. Synthesis of PANI–GO Composites

In a round-bottomed flask, 50 mL of chloroform and 2.3 mL of DBSA were mixed under constant stirring. Then, 1.5 mL of aniline were added slowly to this mixture followed by dropwise addition of 25 mL of 1.1 M sulfuric acid and 25 mL of 0.09 M ammonium per sulphate (APS). Then, 0.015 g of GO were dispersed in 20 mL of distilled water through sonication for 30 min to form a uniform dispersion. This dispersion was slowly added to the polymerization bath and stirred for 24 h. The green precipitate of the PANI–GO composite was washed 3 times with distilled water and then with acetone to remove unreacted species. This clear precipitate was dried in an oven at 60 °C. The same procedure was repeated to synthesized composites of PANI and GO with a different percent amounts of GO. For comparison, PANI without GO was also synthesized. These samples were coded as mentioned in Table 1.

### 2.4. Characterization

To confirm successful formation of PANI–GO composites, different characterization techniques were employed. In this regard, Fourier transmission infra-red (FTIR) spectroscopy was the first choice to trace the existence of different functional groups in the structure of the composites. FTIR spectra were recorded (scanning over the wavenumber range, 400–4000 cm^−1^, with 2 cm^−1^ resolution) with an IR Affinity-S1 spectrophotometer (Shimadzu, Tokyo, Japan). Elemental mapping and surface imaging of the synthesized samples was carried out through SEM and SEM-EDAX analysis (Helios G4 CX DualBeam microscope equipped with OctaneElite). Nano Measurer 1.2.5 software was used to determine the size of the particles. The Brunauer–Emmett–Teller (BET) surface area was determined in the N_2_ atmosphere through the adsorption–desorption method with a surface area analyzer model 2200 e Quanta Chrome (Quanta Chrome, Boynton Beach, FL, USA). To check the thermal degradation pattern, structural aspects, and thermal stability of the composites, thermogravimetric analysis (TGA) was carried out. It was executed by making use of Perkin Elmer (Waltham, MA, USA) at a heating rate 10 °C/min under an N_2_ atmosphere.

Cyclic voltammetry (CV), Galvanostatics charge discharge analysis (GCD), and potentiostatic electrochemical impedance spectroscopy (EIS) of the synthesized samples were executed in a three electrode cell, containing 1 M H_2_SO_4_, using Reference 3000 ZRA potentiostat/galvanostat (Gamry, Warminster, PA, USA). A saturated calomel electrode (SCE) and coiled wire of gold were utilized as the reference and counter electrode, respectively. Further, 80% PANI–GO composite, 10% activated carbon, and 10% PTFE dispersed in DMF and uniformly drop coated on the gold sheet electrode and after vacuum drying were used as the working electrode.

To check the material for its practical application, a symmetric ultracapacitor device was assembled, using two identical current collectors coated with the synthesized sample. A similar amount of PANI–GO nanocomposite was deposited on both electrodes. Filter paper dipped in 1 M H_2_SO_4_ was used as the separator.

## 3. Results and Discussion

### 3.1. Fourier-Transmission Infrared Spectroscopy

**Figure 1:**
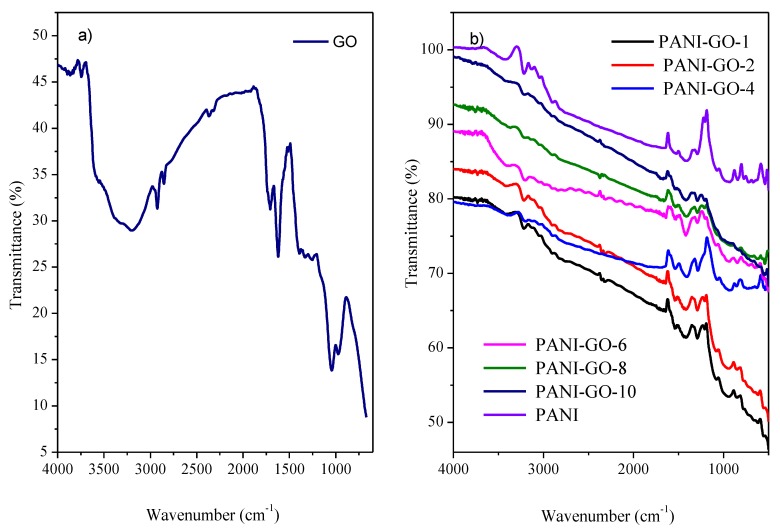
FTIR spectra of (**a**) GO and (**b**), PANI, and PANI–GO composites.

Formation of the PANI–GO composite was confirmed through FTIR spectroscopy. Respective spectra recorded for GO, PANI, and PANI–GO composite are depicted in Figure 1a,b, as indicated. In Figure 1a, a prominent absorption band is positioned at 1726 cm^−1^, most likely referring to C=O stretching type vibration of GO. The band at 1620 cm^−1^ might be designated to aromatic C=C stretching. The bands at 1414, 1241, 1200, and 1049 cm^−1^ are, respectively, correlated with carboxy C–O, carboxy C–OH, epoxy C–O, and alkoxy C–O–C linkages [28]. These functional groups are generally positioned at the margins of the GO sheets. Additionally, O–H stretching type vibration is associated with a characteristic band at 3376 cm^−1^ [29].

The FTIR spectrum of PANI exhibit characteristic bands appearing at 1551/1494 and 1241 cm^−1^ due to C-C stretching of the quinoid/and benzenoid moieties and C=N stretching vibrations of PANI [28]. The in-plane vibration band is observed at 1183 cm^−1^ [30]. The strong absorption bands in the PANI spectrum at 1581 and 1498 cm^−1^ are the consequence of the stretching mode of vibrations of C=C in the quinonoid and benzenoid rings, respectively. The bands appearing, respectively, at 1326 and 1183 cm^−1^ are evidence of the presence of aromatic C‒N, C=N, and C‒H stretching mode of vibrations in the polaron framework of PANI. Bands at 684 and 1231 cm^−1^ are due to symmetric and asymmetric stretching vibrations of the O=S=O and S–O groups of the dopants. The presence of an important band at 1006 cm^−1^ indicates NH^+^…SO^-^_3_ interaction among PANI and dopants [31]. The FTIR spectra of the composites (Figure 1b) depict the emergence of characteristic bands of the carboxyl (COOH) (1724 cm^−1^), O=C‒O (2342 cm^−1^), and OH groups (1064 and 3026–3500 cm^−1^) along with characteristic PANI bands showing the existence of both PANI and GO components in the composite. Additionally, a slight decline in the strengths of the 1726 and 1620 cm^−1^ characteristic bands of GO suggest the production of a PANI layer at its surface. A shift in the characteristic bands of PANI to higher wavenumbers might be attributed to the interlinkage among PANI and GO, suggesting the existence of a conductive composite framework [28,32].

### 3.2. Scanning Electron Microscopy

**Figure 2:**
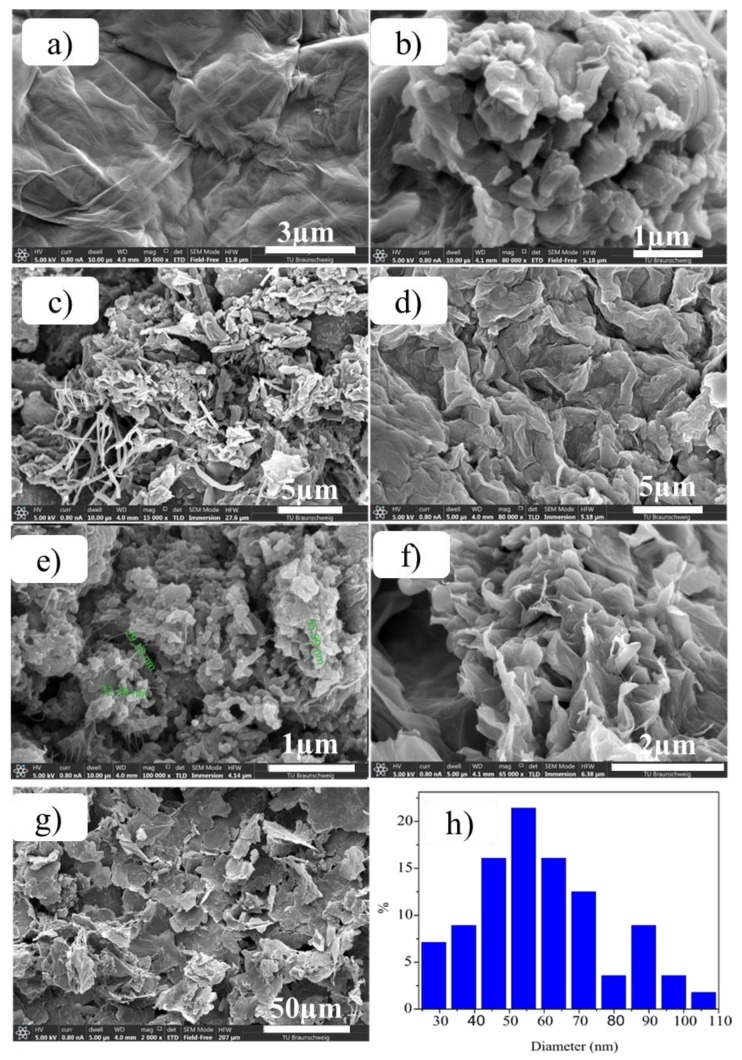
SEM images of (**a**) GO, (**b**) PANI (**c**) PANI–GO-1, (**d**) PANI–GO-4, (**e**) PANI–GO-6, (**f**) PANI–GO 8, (**g**) PANI–GO-10, (**h**) particle size distribution histogram of PANI–GO-6.

Figure 2a depicts the SEM images of GO, exhibiting a typical flake-like morphology of graphene oxide [33]. In Figure 2b–h, SEM images of the PANI–GO composites are showcased. The effect of the changing percent concentration of GO can clearly be observed from these images. It is depicted that PANI assembles on the surface of GO sheets [34]. It seems that DBSA might have played a very effective role in controlling the morphology and uniform growth of the active material on GO [11]. DBSA is also reported to boost the specific capacitance value of the materials [35].

**Scheme 1:**
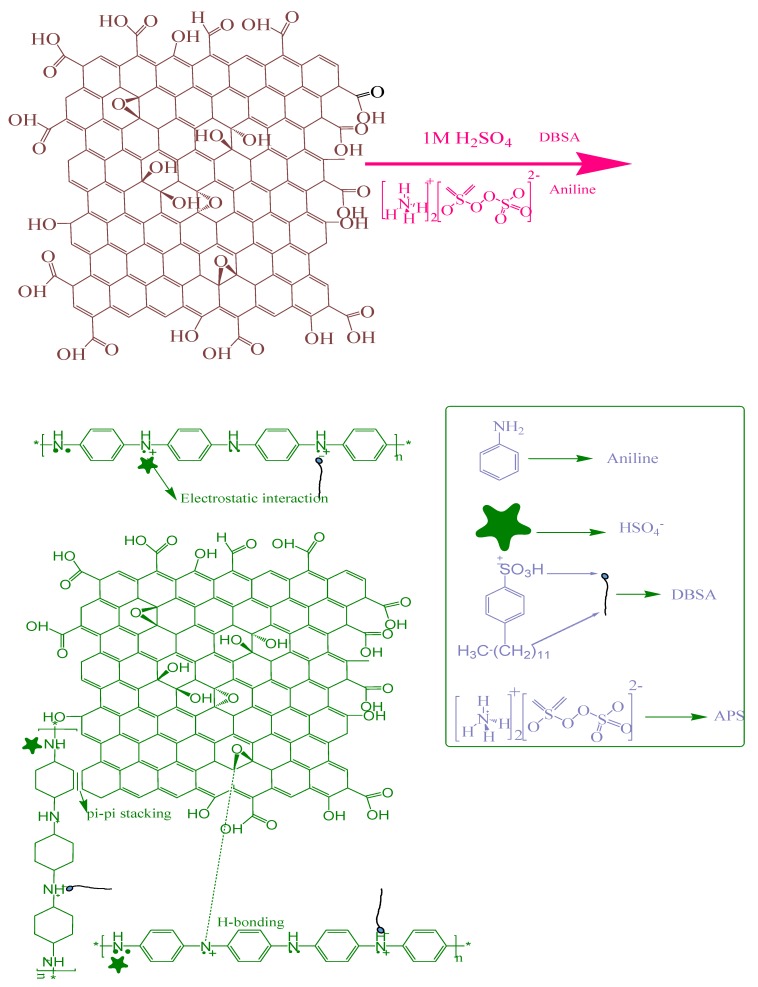
Formation of PANI–GO composites.

It is envisaged that aniline and the dopant molecules are adsorbed on the surface of GO and are prone to oxidization by APS, resulting in the formation of PANI chains [34,36,37,38]. This growth of PANI at the surface of GO sheets is possible because of the enhanced surface area and various oxygen-containing functional groups, which are active sites for polymerization. The APS does not act merely as an oxidant for the process of oxidative polymerization of aniline but also behaves as a surface-directing species to improve the synthesis of PANI on the surface of GO sheets. The PANI backbones are additionally stabilized on the surface of GO via Vander Waals forces and π–π interactions [39]. As DBSA is composed of a hydrophilic group (–SO_3_H) and long hydrophobic chains (CH_3_ (CH_2_)_11_) [35] while the aniline monomer is composed of a hydrophobic benzene-ring and hydrophilic NH_2_ group (amphiphilic molecule) [40], DBSA facilitates the interaction among reactants during polymerization and thus aids in the uniform growth of PANI at the surface of GO sheets in PANI–GO composites. Composite materials are formed because of the electrostatic attraction, π–π forces [41], and hydrogen bonding that takes place among oxygen functional groups of GO and nitrogen atoms of PANI in the –NH– group [42]. A representation of the formation is shown in Scheme 1.

The morphology of these composites is very different from individual components. Variation in morphology takes place with a change in the ratio of raw materials. These prominent changes in morphology reveal that GO is introduced into composites and the morphology can be varied by changing the ratio of individual components to obtain a desired electrochemical performance [43]. At the 6% of GO in the reactor bath, highly porous nanostructures with distributed nanofibers are formed (Figure 2e). Particle size calculation was done for this sample by taking 56 selected particles from Figure 2e and plotting the diameter vs. frequency (Figure 2h). The maximum, mean, and minimum values of the particle sizes were found to be 105.7, 28.3, and 59.57 nm, respectively. From now onward, the sample PANI–GO-6 will be termed as PANI–GO-nanocomposite.

### 3.3. Elemental Analysis and Mapping of Phases

**Figure 3:**
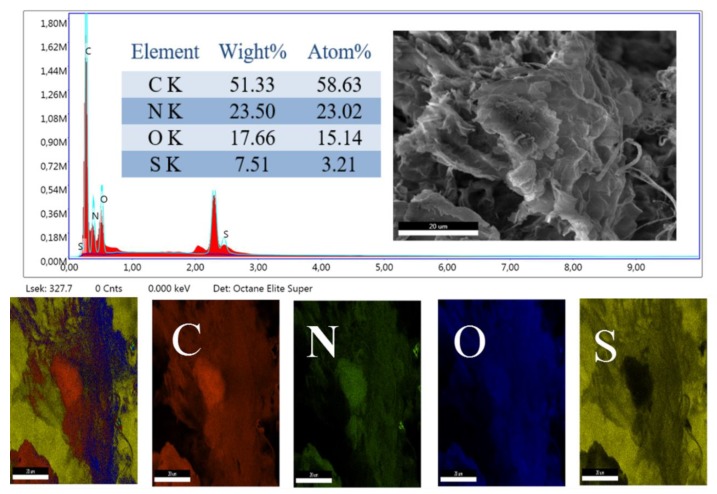
Elemental analysis and mapping of PANI–GO-nanocomposite.

In order to find different elements and their distribution in the synthesized samples, energy dispersive X-ray (EDX) analysis was accomplished (Figure 3 and Figure S1). In Figure S1a, the EDX spectrum of GO shows C and O as the main component while the composite materials showcase all the elements of GO and PANI, including C, O, and N. The % weight of N and O atoms, respectively, decreases and increases with an increasing GO percentage. The presence of a high amount of S in the synthesized samples suggests effective incorporation of the dopants.

### 3.4. Thermogravimetry Analysis

Thermogravimetry analysis (TGA) is a suitable and very useful technique for the determination of dopants and water content in polymers. Beside this, it is useful to determine the thermal degradation pattern, structural aspects, and thermal stability [44]. The degraded matter at each step of the curve can be determined through the mass loss profile.

To explore the extent to which GO, PANI, and PANI–GO composites are thermally stable, TGA analysis was executed, with the thermograms depicted in Figure S2. The weight loss of GO starts before 100 °C, which is because of the removal of physically adsorbed water [45]. The weight loss of GO is 45% at 251 °C. This might be due to pyrolysis of the labile oxygen-containing functional groups present in GO.

In Table S1, the weight loss steps of GO, PANI, and PANI–GO composites are given. PANI mostly manifests as three steps of decomposition. The decomposition prior to 260 °C is mainly because of the deprivation of the moisture content and dopant. The PANI backbone begins to break down at temperatures above 260 °C.

The first weight loss step of PANI–GO composites is attributed to the elimination of moisture. The second weight loss step is due to the removal of dopant molecules [46]. PANI–GO shows a rapid mass loss at around 300 °C due to the decomposition of oxygen-containing groups, such as –OH, –CO–, and –COOH [47].

However, as can be seen from Figure S2, the thermal stability for the nanocomposite is shifted in the direction of elevated temperatures in comparison to PANI and GO. Complete decomposition of PANI and GO occurs at 639 and 615 °C., respectively, while for the PANI–GO-nanocomposite, it is shifted to 825 °C (Table S1). This enhanced thermal stability of the nanocomposites might be assigned to the covalent bonding among the GO and PANI backbone [48]. Additionally, the covalent bonding also results in a substantial π–π stacking force among the basal plane of GO and the PANI backbone [49].

### 3.5. Electrochemical Study

To investigate the electrochemical performance of the synthesized samples, three different techniques, including cyclic voltammetry, electrochemical impedance spectroscopy (EIS), and galvanostatic charge discharge (GCD) analysis were applied.

#### 3.5.1. Cyclic Voltammetry

**Figure 4:**
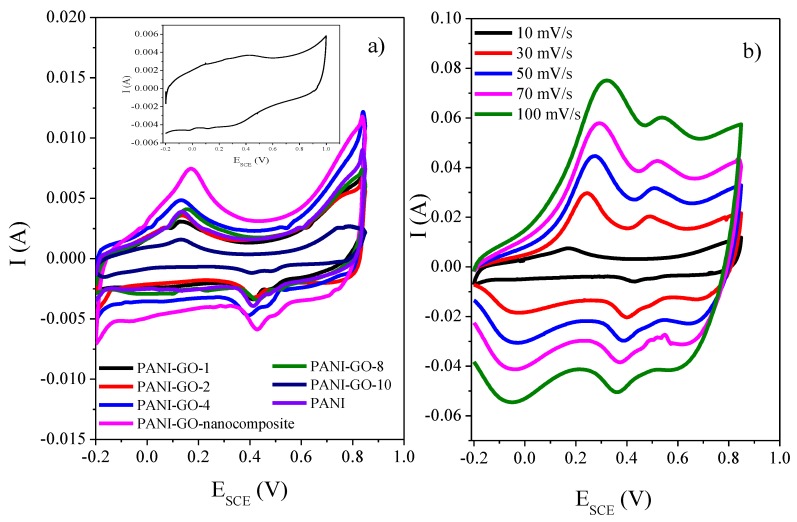
Cyclic voltammogram of (**a**) PANI–GO composites as indicated. (Inset) GO at 10 mV/s and (**b**) PANI–GO-nanocomposite at different scan rates, on the surface of the gold electrode.

Electrochemical assessment of the samples was accomplished in an assembly of three electrodes, where 1 M H_2_SO_4_ was utilized as the aqueous electrolyte. The same amount of the materials was loaded on the surface of the gold electrode. Figure 4a depicts the cyclic voltammograms (CVs) registered in the potential window of −0.2 to 0.85 V for PANI–GO composites at 10 mV/s. Typical peaks owing to redox transformation of PANI can be observed [50]. These peaks are due to leucoemeraldine/emeraldine and emeraldine/pernigraniline transformation of PANI, suggesting that the PANI-like character dominates in the composite material. The cyclic voltammogram of GO was registered at a scan rate of 10 mV/s at the potential window of −0.2 to 0.9 vs. SCE and is presented in the inset of Figure 4a. The CV exhibits an almost rectangular shape, a distinctive behavior of EDL capacitance [51]. It can be seen in Figure 4a that the area enclosed by the CV curve of the PANI–GO-nanocomposite is broader than those of other samples in the frame, suggesting its superior capacitance properties. The capacitance of these composites is chiefly contributed by PANI while GO seems to produce a synergy with PANI, imparting some extra capacitance to the composite material.

In Figure 4b, CV curves of the PANI–GO-nanocomposite at different potential scans (10 to 100 mV/s) are depicted. At higher scans, the specific peak current and response is enhanced while the shape of CV is sustained, representing a good rate capability of the electrode [32,52,53]. These curves exhibit a rectangular shape as well as redox peak pairs, illustrating the concurrence of the electrical double-layer capacitance (EDLC) as well as pseudocapacitance. Curves in CVs are slightly distorted at high potential scans as a consequence of a large time constant (τ = RC) produced by enhanced capacitance and/or resistance and pseudo-reversible kinetics of PANI [54]. The specific capacitance calculated at various scan rates is given in Figure S3.

#### 3.5.2. Galvanostatic Charge Discharge Analysis

**Figure 5:**
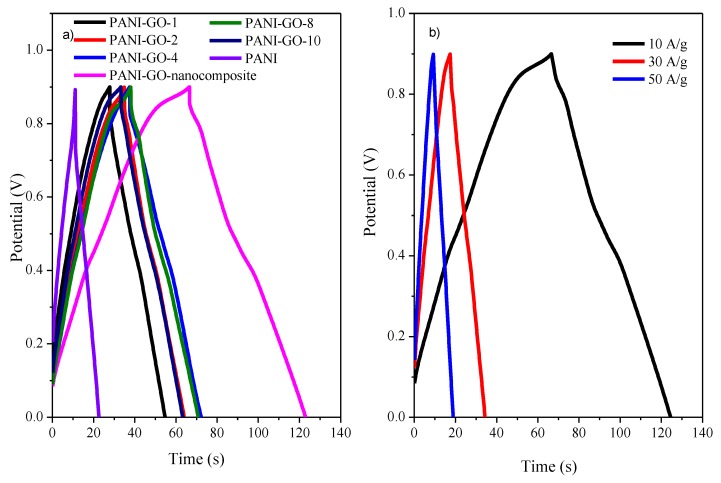
Galvanostatic charge discharge analysis of (**a**) PANI–GO composites at a current density of 10 A/g and (**b**) PANI–GO-nanocomposite at various current densities.

A potential window of 0 to 0.9 V vs. SCE was employed to record the galvanostatic charge discharge (GCD) curves at a current density of 10 A/g (Figure 5a). The specific capacitances were calculated from GCD analysis. These GCD curves show deviation from the perfect triangular shape, manifesting the remarkable participation of pseudocapacitance of PANI in PANI–GO composites [43]. At the commencement of discharge, the IR drop can indicate the internal resistance of the composite material. In the present case, the IR drop is not significant, which shows a lower internal and diffusion resistance of the material [55]. All composites with different percentages of GO possess high capacitance in comparison with PANI and GO alone.

The following equation was applied for the calculation of specific capacitances:

(1) $$C_{s}=\frac{Jt}{\Delta V-IR},$$

(2) $$J=\frac{I}{m},$$

where Δ*V* is the potential window for the GCD process, *I* is the current applied, *t* is the discharge time, and *m* is the mass of the electrode material.

The specific capacitance values, calculated from GCD curves, are presented in Table 2. The specific capacitance value increases with an increasing GO percentage, reaching a maximum of 658 F/g for the nanocomposite, and then decreases further. This trend suggests that the synergy between PANI and GO is at its maximum in the case of the nanocomposite [56]. Charge transfer complex formation between these two individual components is expected to be responsible for the increase in the specific capacitance. The porous structure of the nanomaterial can facilitate electrolyte ion and electron transport to the inner part of the composite materials. It can also be related to the increasing number of active sites for redox reactions as GO contains several hydrophilic functional groups that facilitate smooth ion transportation to the inner material. The decrease in the specific capacitance value for samples with greater GO percentages in the reaction bath might be due to the fact that GO at the surface of electrode results in interfering electron transfer because of its smaller conductivity. A high amount of GO leads to limited double layer charging in the inner electrode material, thus only the outside of the electrode is electro-active, which decreases the overall capacitance [43].

This argument can further be supported by the Brunauer–Emmett–Teller (BET) values of the samples. The technique was used to study the N_2_ adsorption–desorption isotherms and the pore size distribution of the synthesized composites (Figure S4). As can be seen from Table 3, with an increase in the GO percentage, the surface area and pore volume increase till the nanocomposite formation takes place at 6% of GO in the sample composition and then decreases beyond this percentage. The pore radius also decreases with the increasing surface area and pore volume. It is expected that a high surface area provides the composites with more active sites on the surface for pseudocapacitance while the large pore volumes of the composites allow rapid electrolyte ion transport and thus contributes to EDLC [45].The highest BET surface area and average pore volume and smallest pore radius of the nanocomposite seems to be responsible for the highest specific capacitance value of this sample.

The specific capacitance value observed for the PANI–GO-nanocomposite is also better than the recently reported values by different researchers, as shown in the comparison provided in Table 4.

The GCD curve for the PANI–GO-nanocomposite at numerous current densities are depicted in Figure 5b and the specific capacitance values calculated from these curves are presented in Table 5. The drop in capacitance with a rise in the current density is the consequence of faradic redox reaction that proceeds for longer time in comparison to EDL capacitance and it cannot compete with the rate of current flow as the current density increases [43] However, the capacitance retention is 85% even at increased current density (50 A/g), thus showing an excellent rate capability due to the synergistic effects of PANI and GO [63]. The IR drop is not significant, which is because of the fact that GO is covalently connected to PANI, which promotes electron transfer during the process of interfacial redox reaction, thus reducing the energy loss [64].

#### 3.5.3. Electrochemical Impedance Spectroscopy (EIS)

**Figure 6:**
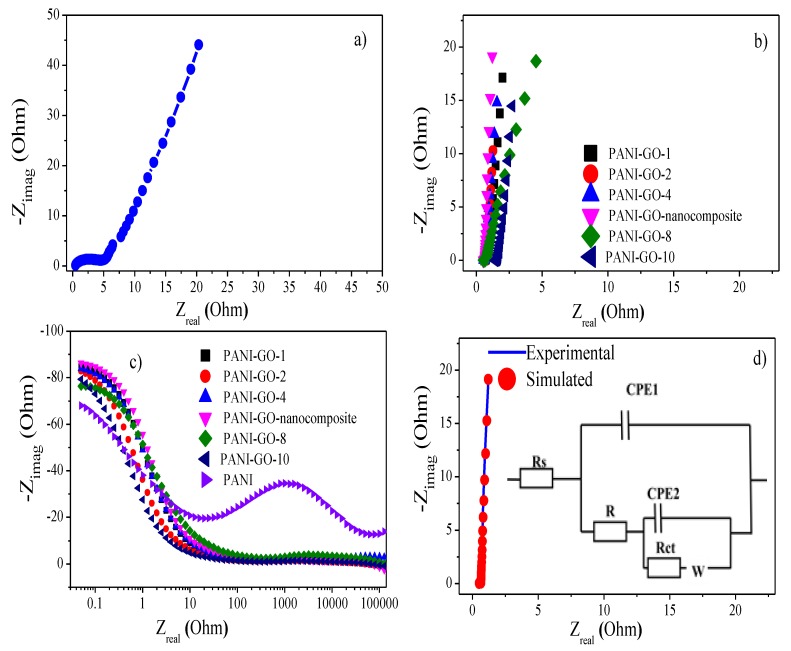
Nyquist plot for (**a**) PANI, (**b**) PANI–GO composites, (**c**) Bode plot of PANI–GO composites, (**d**) Experimental and simulated plots for nanocomposite PANI–GO-nanocomposite.

EIS analysis is significant for the purpose of exploring the interior resistance of the electrode material. To acquire a detailed knowledge of the capacitive characteristics of the PANI–GO composites, an EIS analysis was accomplished, and the results are given in Figure 6. It is a useful technique to explore the electrochemical behavior material in its bulk and interface of its electrode and electrolyte. It is an essential analytical technique employed to obtain knowledge about the frequency responses of ultracapacitors and the type capacitive phenomena that take place at the electrode [65].

The impedance test was executed in the frequency range of 0.05 Hz to 100 kHz at open-circuit potential, as depicted in Figure 6a,b, for PANI and PANI–GO composites. The Nyquist plot is composed of a depressed semicircle in the region of high frequency and a vertical line in the region of low frequency. The smaller semicircle indicates excellent electrical conductivity and a lower resistance value of the composite material [16,66].

Equivalent series resistance (ESR) is displayed by capacitors, which contain bulk solution resistance, Rs, along with electrode internal resistance [67]. From the Nyquist plot, the ESR might be achieved, at the end of high frequency, where the semicircle cuts on the real axis, where Rct is the charge-transfer resistance acquired from the radius of the arc at the real axis.

GO can serve new electroactive sites and it also reduces the ion diffusion pathway, resulting in its quick conduction. The low Rct is beneficial for quick charge transfer and results in enhanced charge storage [68]. As observed in the SEM images, these composites possess a porous structure and very small particle size compared to PANI without GO. This smaller particle size is very useful for efficient charge transfer. The PANI prepared without GO possesses capacitance that is rather low because of the massive Rct value. Very small Rs and Rct values are indications of a fast charge–discharge phenomenon, a reduction in values of ESR, and excellent electrical conductivity of the electrode/electrolyte. Fast ion diffusion and lower resistance might be due to the enlarged specific surface area and also the porous structure of these composites [45].

In AC impedance, the nearly vertical arm in the region of low frequency depicts an outstanding capacitive characteristic, which represents very quick diffusion of ions and adsorption in/on the electrode material [45]. In the lower frequency region, the impedance curve exhibits the trend to be parallel to the imaginary axis, revealing the capacitance characteristic. The diversion in slope may be due to pseudocapacitance of PANI. The Rs, Rct, and ESR values determined from Nyquist plots in Figure 6a,b are given in Table 6. All the samples have lower values of Rs, Rct, and ESR and thus better electrochemical performance. Among all the samples, the nanocomposite PANI–GO has the lowest values of Rs (0.521 Ω) and ESR (0.661 Ω).

The phase angle reliance on the frequency was assessed for the synthesized materials and is depicted in Figure 6c. The knee frequency (*f*_0_) is the particular frequency where the phase angle attains a value of 45°. At this frequency, the resistive and capacitive impedances are equivalent. Following this point, the materials depict greater resistive characteristic at higher frequencies, where the relaxation time (τ_0_ = *f*_0_^−1^) stipulates the smallest time necessary to discharge the whole energy with an efficiency larger than 50%. It is typical that a higher knee frequency correlates with a higher rate capability and lower to.

The Bode diagram (Figure 6c) depicts that the phase angle values are 83° for PANI–GO-1 and PANI–GO-2; 83.96° for PANI–GO-4; 86.34° for PANI–GO-nanocomposite; and 78°, 79°, and 65° for PANI–GO-8, PANI–GO-10, and PANI, respectively. The phase angle is 86.34° for the nanocomposite, which is very near to an ideal capacitor. The slope of the lines at low frequencies declares the capacitive behavior. Certainly, smaller values of resistance manifest higher electrical conductivity inside the whole inner structure. Table 6 shows the frequency at *f*_0_ and that corresponding to [69]. It is clear from Table 7 that PANIGO-nanocomposite shows the smallest time constant value of 0.630 s. This lower time constant is the confirmation of quick ion transport and diffusion inside the entire inner material.

The Nyquist plot for PANI-GO-nanocomposite is explained by simulating the experimental EIS curve to an equivalent electrical circuit as depicted in Figure 6d. It is composed of Rs, Rct, n (frequency power), W (Warburg), and CPE (constant phase element). As C = CPE^n^, so CPE is equivalent to a capacitor when n = 1 [70]. The value of n varies from −1 to 1. It is equivalent to an ideal inductor for n = −1. When n = 0, it is an ideal resistor and for n = 0.5, it shows the diffusion characteristic [71]. It emerges as an equivalent circuit from the non-homogeneity, porosity, and nature of the electrode materials. In Figure 6d, two constant phase elements, which are CPE1 and CPE2, are utilized to represent double layer capacitance and pseudocapacitance, respectively [72]. The values for elements of the equivalent circuit are Rs (0.521 Ω), R (0.140 Ω), CPE1 (0.159 S*s^a), n1 (0.983), Rct (0.140 Ω), W (1 × 10^−6^ S*s^(1/2)), CPE2 (3.4 × 10^−3^ S*s^a), and n1 (0.871).

From these results, it can be concluded that the nanocomposite material possesses much better properties than the other samples under study. Therefore, the nanocomposite was selected to further elaborate its applicability in energy storage by fabricating an aqueous symmetric supercapacitor device utilizing gold and copper as current collectors.

### 3.6. Aqueous Symmetric Supercapacitor Device Using Gold as the Current Collector

The electrochemical supercapacitive properties of the nanocomposite coated gold sheet electrodes were investigated for an aqueous symmetric cell using 1 M aqueous H_2_SO_4_ electrolyte and are presented as follows.

#### 3.6.1. Cyclic Voltammetry

**Figure 7:**
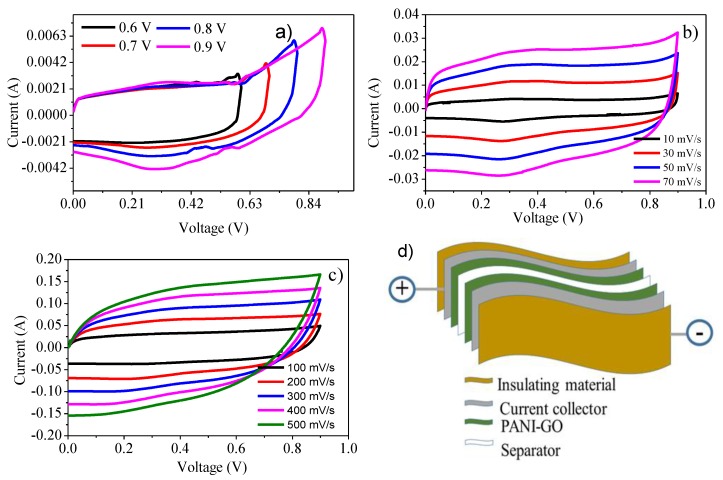
CVs of an aqueous symmetric device, fabricated with PANI–GO-nanocomposite-coated gold sheets, (**a**) at different potential limits, (**b**) at various lower scan rates (10–70 mV/s), and (**c**) at various higher scan rates (100–500 mV/s). (**d**) Configuration of the aqueous symmetric device.

Figure 7a depicts the CVs of the device at a scan rate of 10 mV/s in the potential windows of 0 to 0.6 V to 0 to 0.9 V. The CV maintains nearly a rectangular shape. CVs at multiple scan rates in the potential window of 0 to 9 V are depicted in Figure 7b,c [73]. In these CVs, redox peaks are restrained. The rectangular-shaped CV curves are indicative of capacitive behavior [10,74]. These results are also supported by the following GCD and EIS results, indicating that the material shows an excellent capacitive response.

#### 3.6.2. Galvanostatic Charge Discharge Analysis

**Figure 8:**
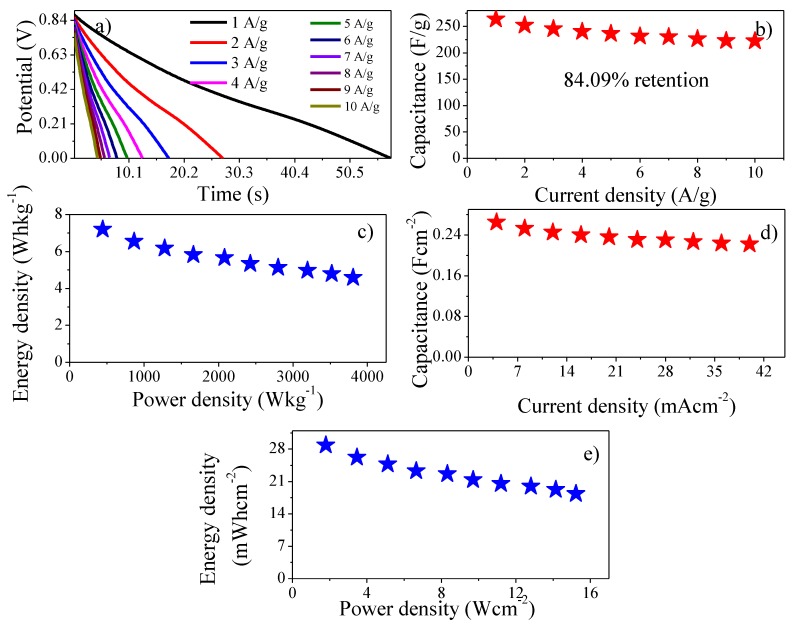
(**a**) Discharge curve of the subject device at various current densities, (**b**) Specific capacitance vs. current densities plot, (**c**) The Ragone plot, (**d**) Dependence of the specific capacitance of the device on the volumetric current density (**e**) The Ragone plot based on the volumetric current density.

The GCD analysis was accomplished in the potential limit from 0 to 0.9 V at current densities of 1 to 10 A/g (Figure 8a). The dependence of the specific capacitance on the current density is presented in Figure 8b. The highest specific capacitance value of 264 F/g was achieved for a current density of 1 A/g. The values decline with increasing current density and reach 222 F/g at 10 A/g. The capacitance retention is 84.09% with an increase in current density from 1 to 10 A/g, showcasing a good rate capability [75]. Moreover, the IR drop is very small, indicating low internal resistance and energy loss [76]. A value of 7.19 Whkg^−1^ of gravimetric energy density and 446 Wkg^−1^ of gravimetric power density was achieved at 1 A/g (Figure 8c). The nanocomposite material showed an areal capacitance of 0.2648 Fcm^−2^ at 4 Acm^−2^, which decreases to 0.222 Fcm^−2^ at 40 Acm^−2^ (Figure 8d). The areal energy density and power density values were, respectively, 28.87 mWhcm^−2^ and 1.79 Wcm^−2^ (Figure 8e).

#### 3.6.3. Electrochemical Impedance Spectroscopy

**Figure 9:**
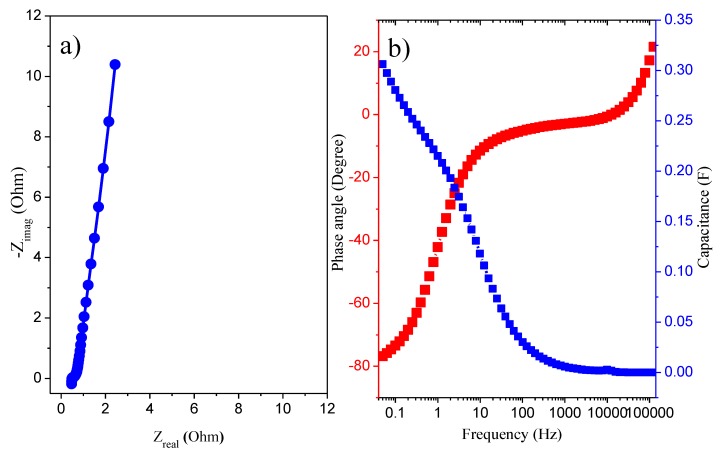
(**a**) Nyquist and (**b**) Bode plot for the device.

Figure 9a shows the Nyquist plot for the symmetric device. From the fitted model, Rs is 0.505 Ω, ESR is 0.726 Ω while Rct is 0.221 Ω. Figure 9b represents the Bode plot, where the phase angle is 76° and shows capacitive behavior. At a phase angle of −45°, the frequency is 0.794 Hz and 1.25 s. This smaller value is evidence of quick ion diffusion, transport inside the entire inner structures, and high electrical conductively of the electrode material due to incorporation of GO [69,77].

#### 3.6.4. Cycling Stability

**Figure 10:**
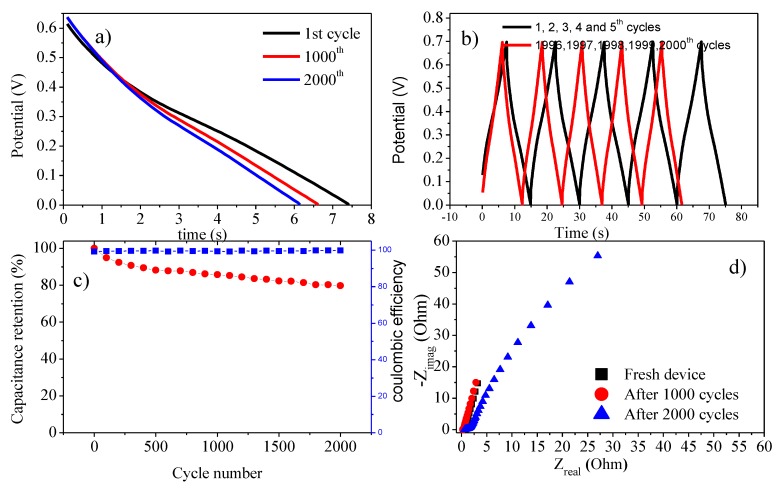
(**a**) Discharge curves for the 1st, 1000th, and 2000th cycles, (**b**) GCD curves for the 1st, 2nd, 3rd, 4th, 5th, 1996th, 1997th, 1998th, 1999th, and 2000th cycles, (**c**) Cycling stability of the symmetric device, (**d**) Nyquist plots recorded for the fresh device after 1000 and 2000 cycles.

Cycling stability is another important feature of supercapacitive devices. To test the material for its stability during charge and discharge, GCD analysis was accomplished in the voltage limit of 0 to 0.7 V for 2000 cycles (Figure 10a). Figure 10b represents the GCD cycles for the first and last five cycles. Figure 10c show that the capacitance retention is 79.83% while the columbic efficiency is 99.83% after 2000 cycles. This decrease in the specific capacitance with charge discharge cycles might be referred to as the destruction of the composite materials in the electrode and/or the mass loss of the electrode after 2000 cycles [78].

To further check the material degradation behavior, EIS was carried for a fresh device and after each 1000 cycles. Figure 10d depicts that the charge transfer resistance decreases after 1000 cycles but increases after 2000 cycles probably due to degradation of the material [78].

### 3.7. Aqueous Symmetric Supercapacitor Device Using Copper as the Current Collector

To check the multifunctionality of the nanocomposite, an aqueous symmetric device was fabricated utilizing copper as the current collector.

#### 3.7.1. Cyclic Voltammetry

**Figure 11:**
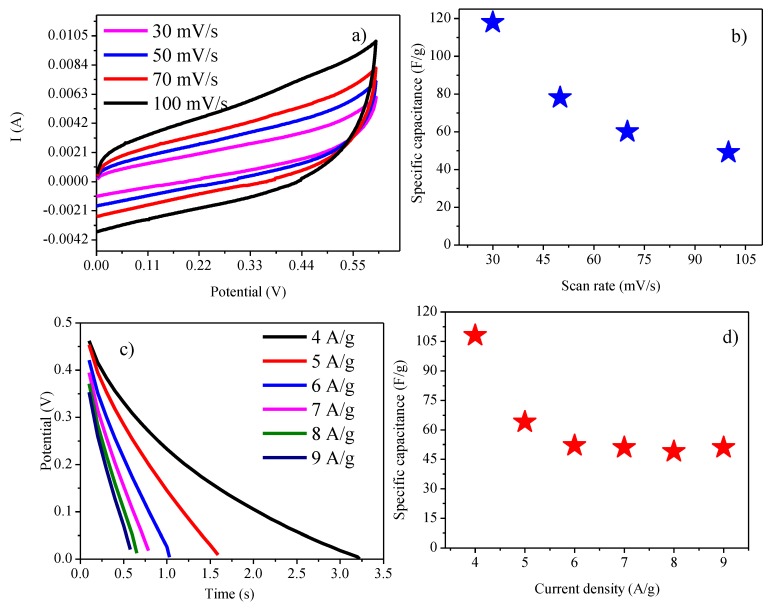
(**a**): CVs of the aqueous symmetric device, fabricated with nanocomposite coated copper sheets, at various scan rates (30, 50, 70, and 100 mV/s), (**b**). Specific capacitance vs. scan rates (**c**). Discharge curves for the device at different current densities, (**d**). Specific capacitance vs. current densities plot.

A symmetric device was fabricated by using copper as the current collector. The device was prepared by depositing an optimized sample on two similar copper current collectors. Filter paper dipped in 1 M H_2_SO_4_ was utilized as the electrolyte. CV was carried out from 0 to 0.6 V at a number of scan rates (30, 50, 70, and 100 mV/s) depicted in Figure 11a.The shape of CV is maintained even at a high scan rate (100 mV/s) while values of the specific capacitance vs. scan rates are given in Figure 11b.

#### 3.7.2. Galvanostatic Charge Discharge Analysis

GCD was carried out at potential limit from 0 to 0.6 V at different current densities (Figure 11c). Specific capacitance vs. current densities are given in Figure 11d. The good performance of the nanocomposite on the copper electrode shows the multifunctionality of the synthesized nanocomposite.

#### 3.7.3. Electrochemical Impedance Spectroscopy of the Symmetric Device Using Copper as Current Collectors

**Figure 12:**
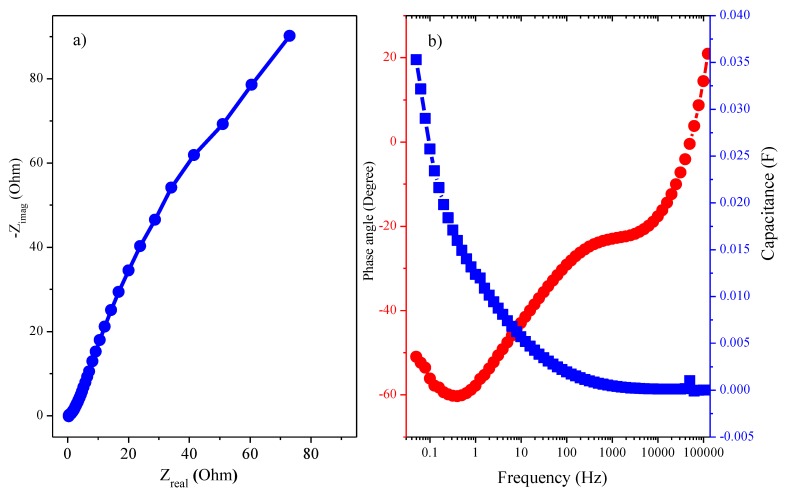
(**a**) Nyquist plot of thee symmetric device using copper as current collectors, (**b**) Bode plot of the symmetric device using copper as current collectors.

To further check the performance of the PANI–GO-nanocomposite as the electrode material when copper was used as the current collector, EIS was carried out. EIS was carried out at a frequency range from 0.05 to 100,000 Hz (Figure 12a). The Nyquist plot shows capacitive behavior, with a solution resistance value of 0.347 Ω and charge transfer resistance of 683 Ω.

The value of n is greater than 0.5 while that of the phase angle is −51° (Figure 12b) [71]. These results show the multifunctionality of the synthesized nanocomposite as it can also be utilized as an electrode material when non-expensive copper is used as the current collectors.

## 4. Conclusions

The synthesis protocol is very important for defining different properties of materials. When properly designed and optimized, materials with valuable properties can be tailored. This is true in the case of the present study as when we optimized the reaction parameters for in situ chemical oxidative synthesis of PANI–GO composites, nanocomposites were obtained in particular conditions. These nanocomposites showed enhanced capacitive and electrochemical properties, an excellent rate capability, and a lower ESR value. EIS analysis showed low Rct, ESR, to, and Rs. A phase angle value close to 90° was observed in certain conditions, which is very close to an ideal capacitor. These nanocomposite materials were active as an electrode material when utilized in the fabrication of symmetric supercapacitor devices with gold or copper as the current collectors, thus indicating their multifunctional abilities.

## Figures and Tables

**Table 1:** Sample codes on the basis of the Graphene Oxide (GO) percent amount.

S. No	Sample Code	DBSA (mL)	H_2_SO_4_ (M)	GO Amount (%)
1	PANI-GO-1	2.3	1.1	1
2	PANI-GO-2	2.3	1.1	2
3	PANI-GO-4	2.3	1.1	4
4	PANI-GO-6	2.3	1.1	6
5	PANI-GO-8	2.3	1.1	8
6	PANI-GO-10	2.3	1.1	10
7	PANI	2.3	1.1	0

**Table 2:** Specific capacitance of the synthesized PANI–GO composites.

S. No	Sample Name	Specific Capacitance Value at 10 A/g (F/g)
1	PANI-GO-1	323
2	PANI-GO-2	345
3	PANI-GO-4	417
4	PANI-GO-nanocomposite	658
5	PANI-GO-8	387
6	PANI-GO-10	355
7	PANI	158

**Table 3:** Surface area, average pore volume, and pore radius.

Sample Name	BET Surface Area (m^2^/g)	Average Pore Volume (cc/g)	Average Pore Radius (Å)
PANI-GO-1	9.865	0.010	21.022
PANI-GO-2	19.067	0.019	19.374
PANI-GO-4	30.664	0.027	17.387
PANI-GO-nanocomposite	39.595	0.037	14.561
PANI-GO-8	6.086	0.005	15.717
PANI-GO-10	4.307	0.004	18.370
PNAI	1.998	0.002	15.768

**Table 4:** Comparison of the specific capacitance value of PANI–GO-nanocomposite with the literature.

S. No	Material Used	Specific Capacitance (F g^−1^)	Current Density (A g^−1^)	Reference
1	Graphene/polyaniline composite hydrogels	180	10	[57]
2	Graphene/PANI nanofiber composites	645	10	[58]
3	PANI/GO/Cu composite	372.1	10	[59]
4	PANI/rGO-HT	320	10	[60]
5	H-NrGO/PANI-10	440	10	[61]
6	PANI/SG	572.2	10	[62]
7	PANI-GO-nanocomposite	658	10	Present work

**Table 5:** Specific capacitance of PANI–GO-nanocomposite at various current densities.

S. No	Current Density (A/g)	Specific Capacitance (F/g)
1	10	658
2	30	597
3	50	561

**Table 6:** Rs, Rct, and ESR for PANI and PANI–GO composites.

S.NO	Sample	Rs/Ohm	Rct/Ohm	ESR/Ohm
1	PANI-GO-1	0.621	0.410	0.761
2	PANI-GO-2	0.669	0.092	0.761
3	PANI-GO-4	0.540	0.129	0.669
4	PANI-GO-nanocomposite	0.521	0.140	0.661
5	PANI-GO-8	0.542	0.182	0.724
6	PANI-GO-10	1.43	0.180	1.61
7	PANI	0.468	4.412	4.88

**Table 7:** Time constant for PANI–GO composites.

S. No	Sample Name	Frequency (*f*_0_), Hz	Time Constant (s)
1	PANI-GO-1	1.267	0.789
2	PANI-GO-2	0.633	1.579
3	PANI-GO-4	1.267	0.789
4	PANI-GO-nanocomposite	1.585	0.630
5	PANI-GO-8	1.267	0.789
6	PANI-GO-10	0.504	1.98
7	PANI	0.504	1.98

## Supplementary Materials

The following are available online at https://www.mdpi.com/2079-4991/10/1/118/s1, Figure S1: Elemental analysis and mapping of (a) GO, (b) PANI, (c) PANI-GO-1, (d) PANI-GO-4, (e) PANI-GO-nanocomposite, (f) PANI-GO-8, (g) PANI-GO-10, Figure S2: Thermo Gravimetric Analysis curves of GO, PANI and PANI-GO composites, Table S1: Weight loss steps of GO, PANI and PANI-GO composites, Figure S3: Specific capacitance vs scan rates, Figure S4: Brunauer–Emmett–Teller (BET) surface area.
