# Supplementary material for: Study on Direct Synthesis of Energy Efficient Multifunctional Polyaniline–Graphene Oxide Nanocomposite and Its Application in Aqueous Symmetric Supercapacitor Devices
Source: Nanomaterials (Basel). 2020 Jan 8;10(1):118. doi: 10.3390/nano10010118 (PMC7022722; doi:10.3390/nano10010118)
Supplement: Supplementary file 1 [file nanomaterials-10-00118-s001.pdf]

Study on Direct Synthesis of Energy Efficient Multifunctional Polyaniline–Graphene Oxide Nanocomposite and Its Application in Aqueous Symmetric Supercapacitor Devices

Hajera Gul [1], Anwar-ul-Haq Ali Shah [2], Ulrike Krewer [3] and Salma Bilal [1,3,*]

[1] National Centre of Excellence in Physical Chemistry, University of Peshawar, Peshawar 25120, Pakistan; hajeragul11@yahoo.com

[2] Institute of Chemical Sciences, University of Peshawar, Peshawar 25120, Pakistan; anwarulhaqalishah@uop.edu.pk

[3] TU Braunschweig Institute of Energy and Process System Engineering, 38106 Braunschweig, Germany;

* Correspondence: s.bilal@tu-braunschweig.de; Tel.: +0049-531-391-63651/3030

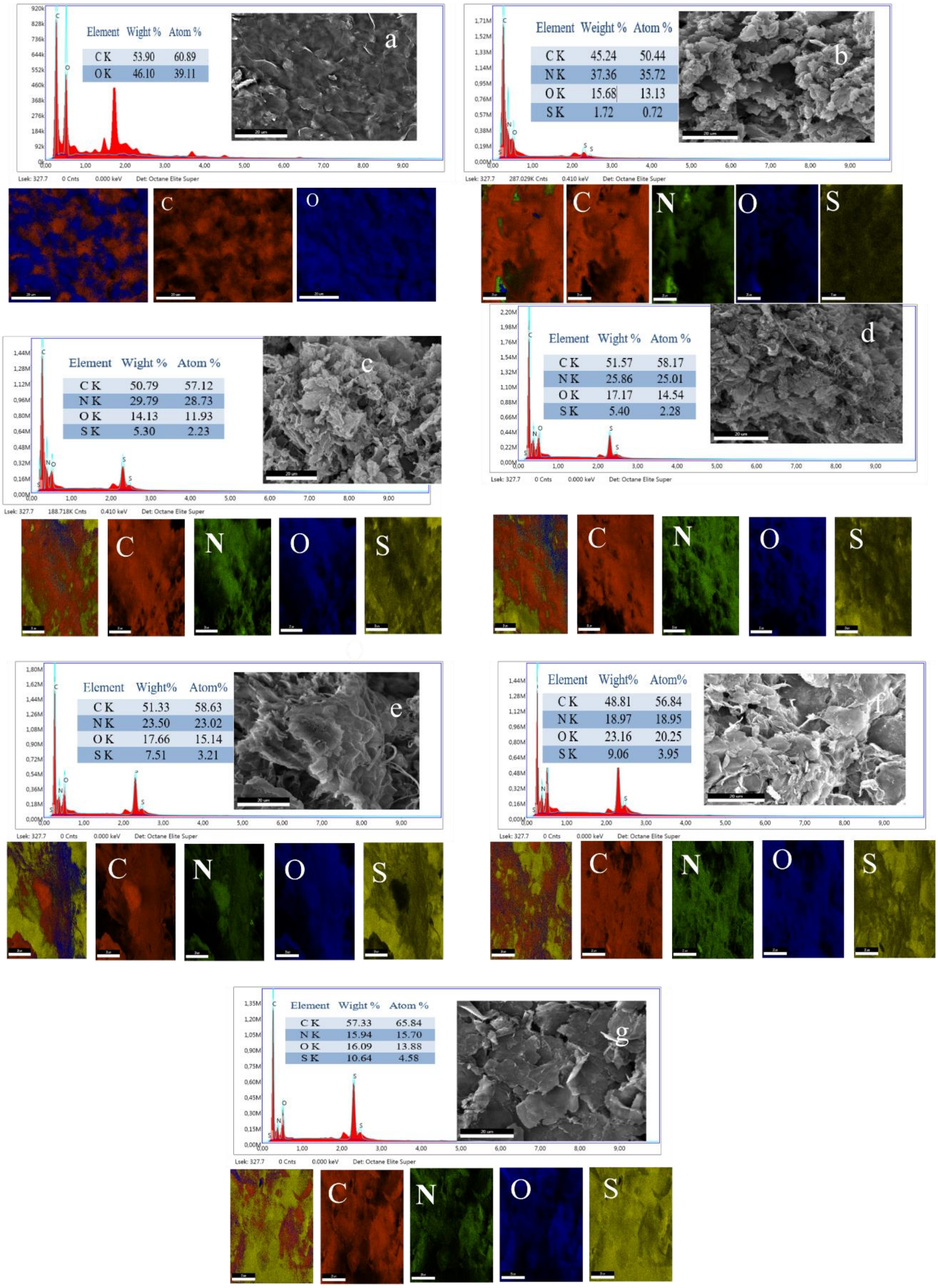

Figure S1. Elemental analysis and mapping of a) GO, b) PANI, c) PANI-GO-1, d) PANI-GO-4, e) PANI-GO-nanocomposite, f) PANI-GO-8, g) PANI-GO-10.

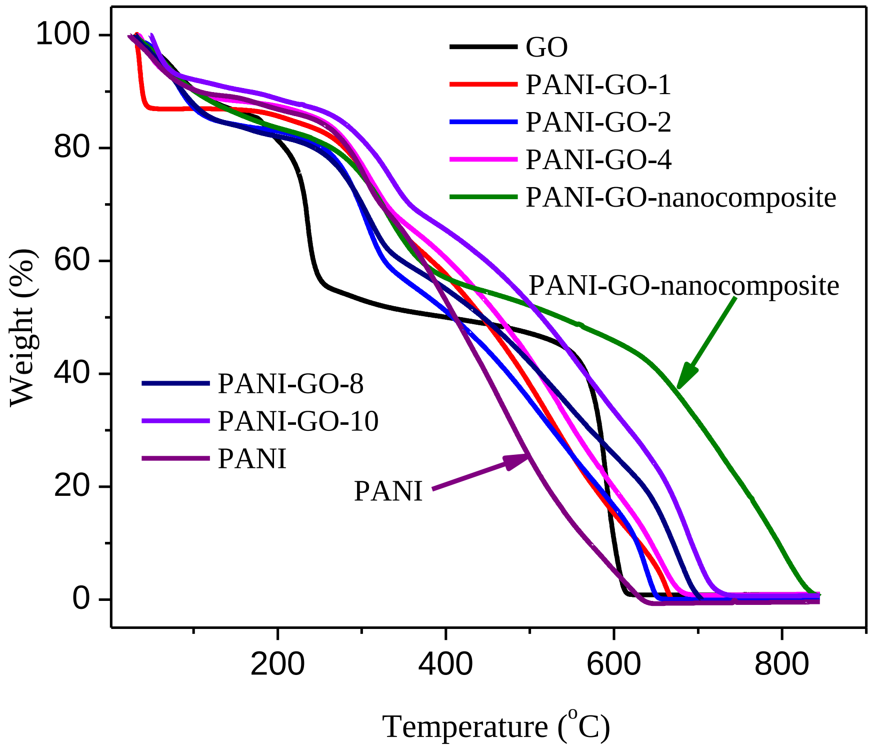

Figure S2. Thermo Gravimetric Analysis curves of GO, PANI and PANI-GO composites.

Table 1. Weight loss steps of GO, PANI and PANI-GO composites.

Sample	Up to temperature ^0C (% weight loss)			
	**First step**	**Second step**	**Third step**	**Fourth step**
GO	191(17)	251(28)	541(11)	615(43.22)
PANI	94(10)	260(6)	639(84)	
PANI-GO-1	44(13)	257(5)	333(17)	664 (64.22)
PANI-GO-2	97(15)	242(4)	319(20)	649(59.7)
PANI-GO-4	80(9)	254(6)	331(19)	678(64.5)
PANI-GO-nanocomposite	97(11)	265(9)	351(29)	825(58.62)
PANI-GO-8	109(14)	248(7)	328(17)	729(61.22)
PANI-GO-10	71(6)	284(8)	351(15)	615(69)

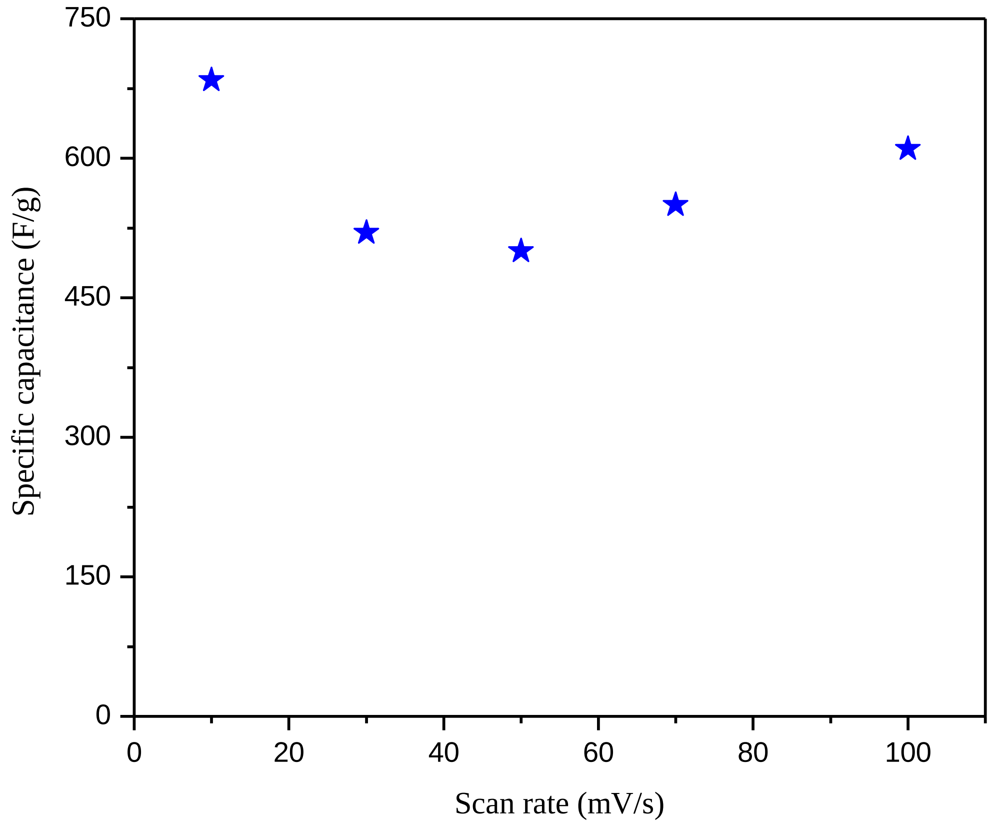

Figure S3. Specific capacitance vs scan rates.

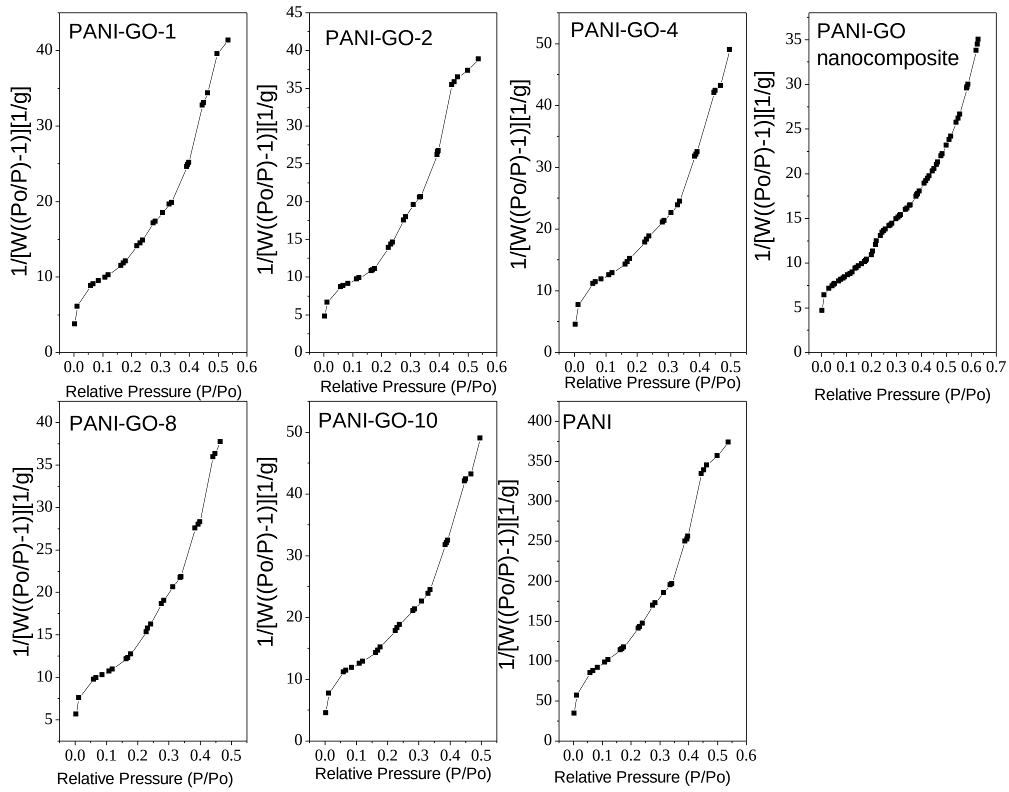

Figure S4. Brunauer–Emmett–Teller (BET) surface area.
